# Development and psychometric evaluation of nurse’s intention to care for patients with infectious disease scale: an exploratory sequential mixed method study

**DOI:** 10.1186/s12912-023-01669-z

**Published:** 2024-01-24

**Authors:** Esmaeil Hoseinzadeh, Hamid Sharif-Nia, Tahereh Ashktorab, Abbas Ebadi

**Affiliations:** 1 Department of Nursing, Faculty of Nursing and Midwifery, Tehran Medical Sciences, Islamic Azad University, Tehran, Iran; 2 Psychosomatic Research Center, Mazandaran University of Medical Sciences, Sari, Iran; 3 Department of Nursing, Amol Faculty of Nursing and Midwifery, Mazandaran University of Medical Sciences, Sari, Iran; 4 Behavioral Sciences Research Center, Life Style Institute, Baqiyatallah University of Medical Sciences, Tehran, Iran; 5 Nursing Faculty, Baqiyatallah University of Medical Sciences, Tehran, Iran

**Keywords:** Intention, Nursing care, Infectious disease, Validity, Realibility

## Abstract

**Aims:**

Nurses who care for patients with infectious disease may experince significant stress and negative psychological reactions. The intention of nurses to care is a complex and multifaceted concept that is influenced by a range of factors. Therefore, this study was conducted to explain the concept of nurses’ intention to care for patients with infectious disease and then develop a reliable and valid scale to measure this concept accurately in Iranian nurses.

**Design:**

This study is a cross-sectional study with a sequential-exploratory mixed-method approach from May 2022 to July 2023. The concept of nurses’ intent to care for patients with infectious disease was explain using deductive content analysis, and item pools were generated. In the sconed step the samples were 455 nurses. Data was collected by an online form questionnaire using a convenience sampling technique. In this step to determine the psychometric properties of nurse’s intention to care for patients with infectious disease scale (NICPS), face and content validity performed. Then construct validity was determined and confirmed using exploratory and confirmatory factor analysis followed by convergent and divergent validity respectively. Finally, scale reliability including stability and internal consistency were evaluated.

**Results:**

The finding showed that NICPS with seventeen items were classified into three factors namely “Social support” with seven items, “Spiritual motivation” with six items and “Job satisfaction” with four items. These three factors explained 56.14% of the total variance. The fit indices showed that the model has a fit and acceptable (TLI, CFI, IFI > 0.9; PNFI, PCFI > 0.5, REMSEA > 0.049, CMIN/DF = 2.477). Reliability revealed acceptable internal consistency and stability (> 0.7).

**Conclusion:**

The finding showed that NICPS has three factors in Iranian nurses. Nursing managers can use these results to provide training and support intervention for nurses in order to increase their intention to care for this patient. Also, the NICPS is a reliable and valid for evaluating this concept in future studies.

**Supplementary Information:**

The online version contains supplementary material available at 10.1186/s12912-023-01669-z.

## Introduction

The term “caring” is defined as the act of experiencing and exhibiting concern, empathy towards others, a sense of dedication and accountability [1], and is recognized as a crucial component of nursing practice [2]. The provision of nursing care is an indispensable element of healthcare services [3].

The constant social, political, and ecological changes that characterize the 21st century have exposed more people to life-threatening acute and chronic infections [4]. Infectious diseases such as Ebola, Zika, Middle East Respiratory Syndrome, Severe Acute Respiratory Syndrome, and influenza, along with other known and unknown pathogens, not only threaten human health but also economic and social well-being [5].

In recent years, the global community has experienced numerous epidemics of infectious diseases, including infectious diseases of influenza A (H1N1) with a new swine origin in Mexico, Ebola in Guinea, MERS in Saudi Arabia, and SARS in China [6], but it is worth noting that Iran has not been heavily affected by these epidemics in the past. However, when COVID-19 was first identified in China in December 2019 and declared a pandemic by the World Health Organization in early March 2020 [7], it placed immense pressure on healthcare systems in Iran and other countries [8]. The pandemic’s destructive and multifaceted effects impacted all aspects of people’s lives [9], leading to the closure of educational and non-essential business activities for several months [10].

During outbreaks of infectious diseases, nurses play a crucial role in caring for patients in infectious disease wards [11]. However, this responsibility can lead to significant stress and negative psychological reactions among nurses, such as depression, anxiety, and irritability. These reactions can be more severe among nurses who unintentionally care for patients with infectious diseases and may result in self-withdrawal from care or leaving the profession [12].

Intention to care refers to nurses’ intention to voluntarily care for patients. Identifying factors that affect nurses’ intention to care is crucial for reducing fear, anxiety, stress, and intention to leave the profession among nurses. It is also essential for improving the quality of nursing care [13].

Examining on nurses’ experiences in caring for patients with infectious diseases show that different themes have been extracted in various countries. These themes include fear of uncertainty, lasting trauma, beyond fear, nonchalance [14], personal protective equipment issues, physical and psychological impact on nurses [15], and health concerns [16]. In Iran, nurses’ experiences showed incomplete preparedness, perceived worst risk, family support, and selfless commitment [17]. The results indicate that depending on the type of infectious disease, nurses’ work situations, and each country’s culture, nurses express different experiences regarding their intentions to care for patients with infectious diseases [14]. Various factors such as job satisfaction, salary satisfaction, organizational commitment, experience in caring for infectious diseases, nurses’ workload, and mental standards play a role in explaining the concept of intention to care for an infectious patient [18]. Therefore, nurses’ intention to care for infectious patients is a multidimensional concept influenced by various factors such as culture, economy, advertising, and religious beliefs [19].

Among the tools designed in relation to the intention of nurses to care for infectious patients is the tool develop to predict the intention of nurses to care for patients with SARS [20] and patients with COVID-19 [21]. Considering the importance of the intention to care for infectious patients, which is the basic pillar of providing nursing services, and considering the cultural, spiritual, and organizational characteristics that affect the intention to care, this study was conducted to clarify the concept of nurses’ intention to care for patients with infectious disease and then develop a reliable and valid scale to measure this concept accurately.

## Method

### Design

This cross-sectional study is going to evaluate the psychometrics of nurses’ intention to care for patients with infectious diseases scale (NICPS) from May 2022 to July 2023. It was completed in two stages: a qualitative stage using a contractual content analysis approach for generating items, and a quantitative stage to evaluate the developed scale’s psychometric properties. The scale was developed for this study and its English language version has been uploaded as a supplementary file.

### Qualitative study and item generation

Twenty-one semi-structured face-to-face interviews with nurses with a mean age of 32.66 years old were conducted in order to fully understand the concept of the nurses’ intent to care for patients with infectious disease, identify relevant structures, and create an item pool. These participants were chosen through purposeful sampling. Every interview lasted 35 to 45 min. The following questions were used in the interviews:

1. *Please explain how you felt about these infectious patients before encountering them, and how do you feel now?*
2. *what made you intent to care of infectious patients?*
3. *What concerns do you have in caring for infectious patients?*
4. *What is your opinion regarding the care of these high-risk patients in terms of disease transmission?*
5. *How is your inner belief about the intention to care?*

In order to get more information and conduct deeper interviews, we used to explore questions like: “What do you mean? Please explain more about this? Or my impression of your words is, have I understood correctly?” At the end of each interview, the recordings were immediately transcribed and processed to a contractual content analysis approach utilizing MAXQDA software version 10. In this stage, 308 initial codes were extracted that were categorized. Then an item pool with 308 item was created using the extracted codes. Due to the large number of items in the item pool, during repeated meetings of the research team, taking into account the adequacy of the number of items for the dimensions of the scale and the necessity of their inclusion, additional and overlapping items were removed and the number of items reached 48. Then, we tried to keep the wording of the items simple, for this reason ambiguous and multipart phrases were removed, which finally left 31 items with a five-point Likert response (1 = Strongly Disagree, 2 = Disagree, 3 = Undecided, 4 = Agree, 5 = Strongly Agree).

### Quantitative study and item reduction

In this step to determine the psychometric properties of NICPS, face validity, content and construct validity, as well as reliability were used. The sample size for each step was different and was explained in each step separately.

### Face validity

To evaluate face validity, two qualitative and quantitative methods were used. In this research, 15 nurses were asked to examine the items of the scale in terms of the level of difficulty, relevancy and ambiguity. In order to perform quantitative face validity, the same 15 nurses were asked to evaluate the importantly of each item by answering to the Likert scale (5 = completely important,4 = somewhat important, 3 = moderately important. 2 = a little important, 1 = not at all important). By calculating each item’s impact score, quantitative face validity was evaluated. The formula impact score = frequency (%)× suitability was used to determine the impact score. The acceptable impact score is more than 1.5 [22].

### Content validity

Content validity of NICPS was evaluated in both qualitative and quantitative ways. The qualitative content validity of NICPS was determined by asking 15 experts with expertise in nursing, psychology, and instrument development to assess the items’ words, grammar, item allocation, and scaling. To check the validity of the content quantitatively, the scale was given to 15 other experts with expertise in nursing, psychology, and instrument development, and the indicators of the content validity ratio (CVR) in terms of essentiality, the content validity index (CVI) and modified kappa index (K*) was measured in terms of relevance [23].

For evaluating CVR each item examined based on a three-part Likert: (1 = not essential, 2 = useful but not essential, 3 = essential). According to Lawshe formula with the number of 15 experts, the minimum acceptable amount of CVR is 0.49. If the resulting score is greater than 0.49, the validity of the content of that item is confirmed, otherwise it will be removed [24]. Following that, modified Kappa (K*) was calculated by asking 15 experts to rate each item’s relevance using a dichotomous response: 1 = relevant, 0 = irrelevant. Kappa values ​​higher than 0.75 are considered excellent [25].

### Item analysis

This stage is the initial evaluation of the tool in the target community and is done before construct validity. At this stage, each item was examined in terms of mean, standard deviation, correlation with other items and the internal consistency of the whole instrument. In connection with examining the correlation between the items, if the items that have a correlation coefficient lower than 0.32 or higher than 0.9 with at least one other item will be removed [26]. At this stage, in order to item analysis, the questionnaire’s online form was created, and its link was sent to 30 nurses via WhatsApp and Telegram.

### Construct validity

#### Participations and samples

In this research, the samples included Iranian nurses who were selected using the convenience method. The study focused on hospital nurses from Golestan University of Medical Sciences. According to the rule of thumb, 200 samples are sufficient for factor analysis [27]. In order to perform exploratory factor analysis (EFA) and confirmatory factor analysis (CFA), a total of 455 nurses were recruited. Online data was collected during this stage. The online scale was made using Google Form and participants received its URL link via email or social media platforms like WhatsApp or Telegram channel.

### Measures

**Fig. 1:**
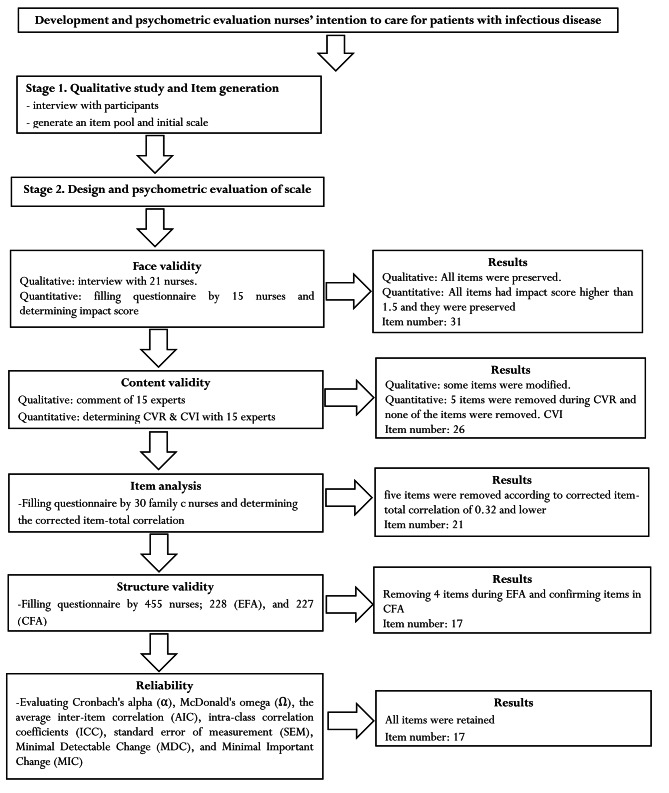
Production phases of nurses’ intention to care for patients with infectious disease scale

At this stage, the used scale contained two parts. The first part was demographic questionnaire such as age, sex, marital status, education level, occupation status, type of responsibility. The second part was NICPS with 21 items to the measuring of the nurses’ intention to care of patients with infectious disease concept with five-point Likert scale response (1 = Strongly Disagree, 2 = Disagree, 3 = Undecided, 4 = Agree, 5 = Strongly Agree). Production phases of NICPS have shown in Fig. 1.

The construct validity of NICPS was evaluated by EFA and CFA. The EFA was evaluated through the maximum likelihood method. According to the correlation results of more than 0.3 between factors, Promax rotation, which is the most common rotation used in humanities studies, was used [28]. Kaiser-Meyer-Olkin (KMO) levels above 0.9 were considered to be excellent. KMO is a statistical test used in factor analysis to determine if the data is suitable for factor analysis. It measures the sampling adequacy of each observed variable in the model as well as the complete model, which is calculated based on the correlation between the variables [26].

In this study, the Horn’s parallel analysis and exploratory graph analysis approaches were used to determine the number of extraction factors. Parallel analysis is a number of correlation matrixes of random variables and is based on sample size and the number of identical variables in real data. In this method, average eigenvalues ​​from random correlation matrixes are compared with eigenvalues ​​from the correlation matrix of real data. In this way, the observed eigenvalue is compared with the second random eigenvalue and so on. Factors corresponding to true eigenvalues ​​that are greater than the average (95th percentile) of parallel random eigenvalues ​​should be extracted, and true eigenvalues ​​less than or equal to the average of parallel random eigenvalues ​​are considered as measurement errors [29].

The critical point for maintaining the item in the factor is considered to be 0.3, which is obtained through the formula: CV = 5.152 ÷ √(n – 2), where CV is the critical value and N is the sample size [30]. Also, items with communalities < 0.2 were removed [31]. At this stage, the structure of the construct obtained through EFA was examined by CFA. The maximum-likelihood approach was used for the CFA. Evaluation of model fit with data was evaluated with the help of standard model fit indices. Among the goodness of Fit, the most common ones were evaluated for the presented model. From the absolute fit index, Chi-square (CMIN), root mean square error of approximation (RMSEA < 0.05), and among the Comparative fit indices, Tucker-Lewis Index (TLI > 0.9), Incremental Fit Index (IFI > 0.9), Comparative Fit Index (CFI > 0.9), and among the parsimonious fit indices, Parsimonious Normed Fit Index (PNFI > 0.5) and Parsimonious Comparative Fit Index (PCFI > 0.5) were calculated, and finally, the ratio of chi-square to degrees of freedom (CMIN/DF < 3) was also evaluated [32].

### Convergent and discriminant validity

The convergent and discriminant validity were assessed using the average variance extracted (AVE), maximum shared squared variance (MSV), and composite reliability (CR). As a result, the criteria for the existence of the convergent validity were AVE > 0.5, CR more than AVE, and MSV less than AVE, while the criteria for the existence of the discriminant validity were MSV less than AVE [33]. Additionally, an innovative approach of Henseler’s Heterotrait-Monotrait Ratio (HTMT) criteria used to assess the discriminant validity. HTMT Ratio < 0.85 was considered as the existence of discriminant validity [34].

### Reliability

In this study, in order to determine the internal consistency of the structure, NICPS was completed by Cronbach’s alpha coefficient, McDonald’s omega coefficient (Ω) and average inter-item correlation (AIC). Alpha and Omega coefficients > 0.7 and AIC 0.2 to 0.4 were considered appropriate. Also, the composite reliability (CR) of the final structure, which is the strongest type of reliability evaluation, and the maximum reliability value (MaxR) > 0.7 was considered acceptable [26].

In this study, the test-retest method was used to check the relative stability. In this way, 25 nurses were asked to complete the scale and it was repeated two weeks later under the same conditions. Then, the agreement between the scores obtained from the two tests was calculated using intraclass correlation coefficient (ICC) and the two-way random-effects model. The value of this index is higher than 0.8, indicating that reliability is acceptable [35]. Absolute reliability was evaluated based on standard Error of measurement(SEM) by the following formula: (SEM = SD_Pooled_ × √(1 − ICC) [36]. The responsiveness was evaluated by calculation the Minimal Detectable Change (MDC) via the formula: MDC95 = SEM×√2 × 1.96 and Minimal Important Change (MIC) via the formula: MIC = 0.5 × SD of the Δ score respectively. It is notable the interpretation of MIC needs to LOA that was estimated using the following formula: LOA = d ± 1.96 × SD difference. Finally, the ceiling and floor effect as well as MDC were evaluated to determine interpretability [37].

### Multivariate normality and outliers

Multivariate outliers were analyzed using Mahalanobis distance p < 0.001, while univariate outliers were evaluated using distribution charts. Additionally, skewness (± 3) and kurtosis (± 7)were used to evaluate the normality of the univariate distribution, and Mardia’s coefficient < 8, was used to check the normality of the multivariate distribution [26].

### Data analysis

Data were analyzed using SPSS/AMOS 27 and JASP0.18.0.0.

### Ethical consideration

The Tehran Islamic Azad University of Medical Sciences Research Ethics Committee approved the protocol of this study (IR.IAU.TMU.REC.1401.116). The purpose of each interview was described to the participants at the start of the interview, and they were then asked for written permission and informed consent to have their answers to questions recorded. They were further reassured that participating to the study was voluntary. Participants received assurances on the confidentiality of their data.

## Results

### Item generation

As a result of the participant interviews, totally seven themes including *job satisfaction, professional ethics, personal Values, standard precautions, preserving health, support, and attitude of patients and their families* were developed. An item pool with 308 items was generated using initial codes. Of these, 31 items were selected as items of the NICPS.

### Item reduction

Based on the results in the qualitative face validity stage, all the items had the necessary clarity and transparency for the respondents. In the quantitative validity stage, all the items had an impact score more than 1.5 and the items were considered suitable for further analysis. In the qualitative content analysis stage, the items were modified based on the recommendations of experts. Based on the results obtained of CVR five items were removed, the total number of the NICPS was reduced from 31 to 26 items. Based on the results obtained of Modified Kappa (K), none of the items were removed. Finally, during the item analysis five items removed and NICPS with 21 items entered the next stage to check the construct validity.

### Demographic profile of participants

Totally 455 nurses with mean ages 30.41(SD = 8.74) and work experience 7.21 (SD = 8.05) years participated in this study. More of nurses were women (74.9%). Most of them were single (54.9%) (Table 1).

**Table 1:** Demographic characteristics of participants (N = 455)

Variables		N (%)
Age		30.41 ± 8.74
Gender	Female	341 (74.9)
	Male	114 (25.1)
Marital status	Single	250 (54.9)
	Married	205 (25.1)
Education level	Bachelor	404 (88.8)
	Master	47 (10.3)
	PhD	4 (9)
Employment status	Employed	184 (40.4)
	Contractual	80 (17.5)
	Design period	191 (42)
Type of responsibility	Nurse	399 (87.7)
	Head nurse	32 (7)
	Supervisor	24 (5.3)
Ward	Emergency	230 (52.9)
	Medical	57 (13.1)
	Infectious	57 (13.1)
	ICU	34 (7.8)
	Others	57 (13.1)
Income	Low	158 (34.7)
	Moderate	245 (53.8)
	High	52 (11.5)
Experience in caring for infectious patients	Yes	404 (88.8)
	No	51 (11.2)
Desire to migrate	Yes	53 (11.6)
	No	402 (88.4)
Desire to leave the job	Yes	199 (43.7)
	No	256 (56.3)
Desire to change job	Yes	168 (36.9)
	No	287 (63.1)

### Construct validity

**Fig. 2:**
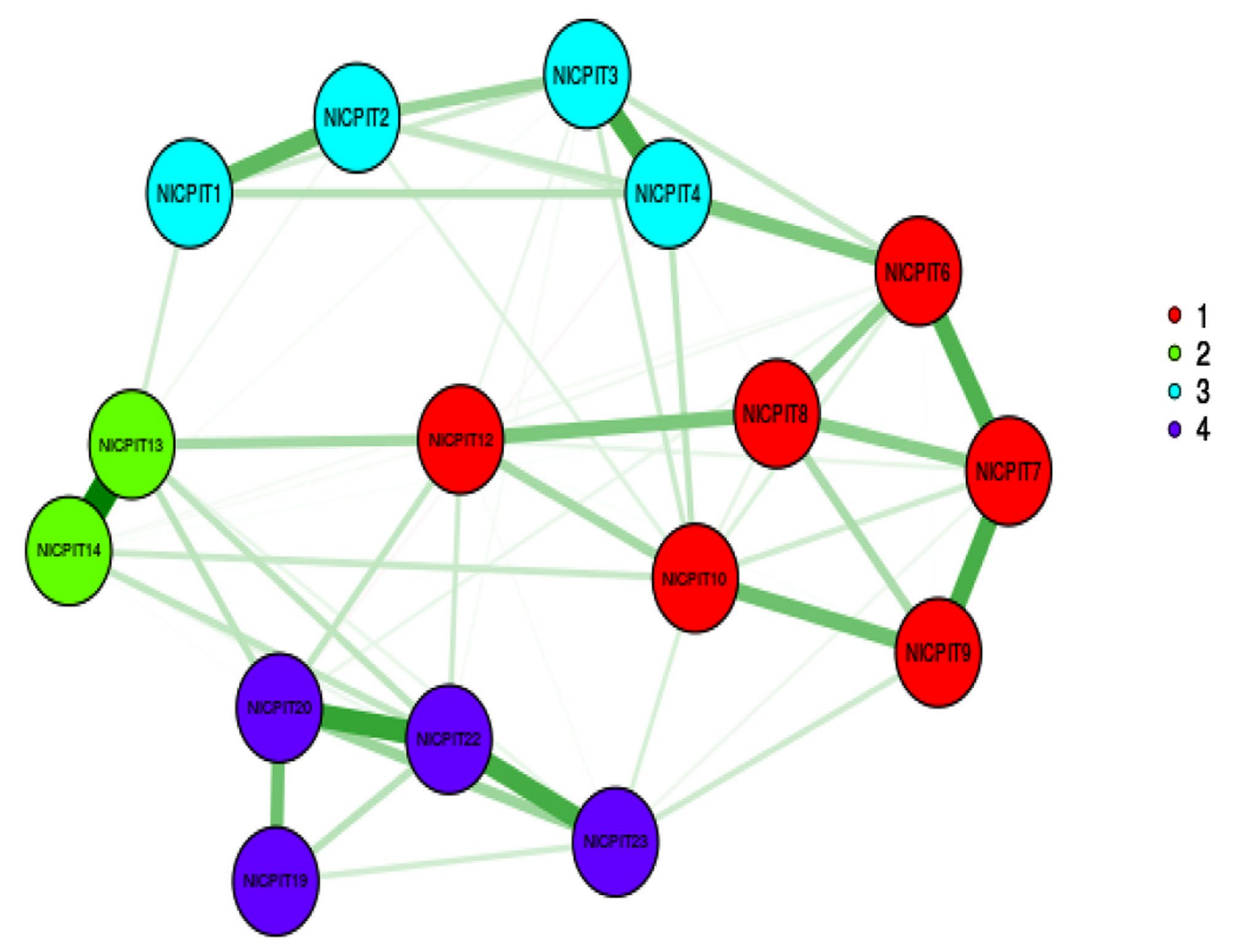
Exploratory graph analysis

**Fig. 3:**
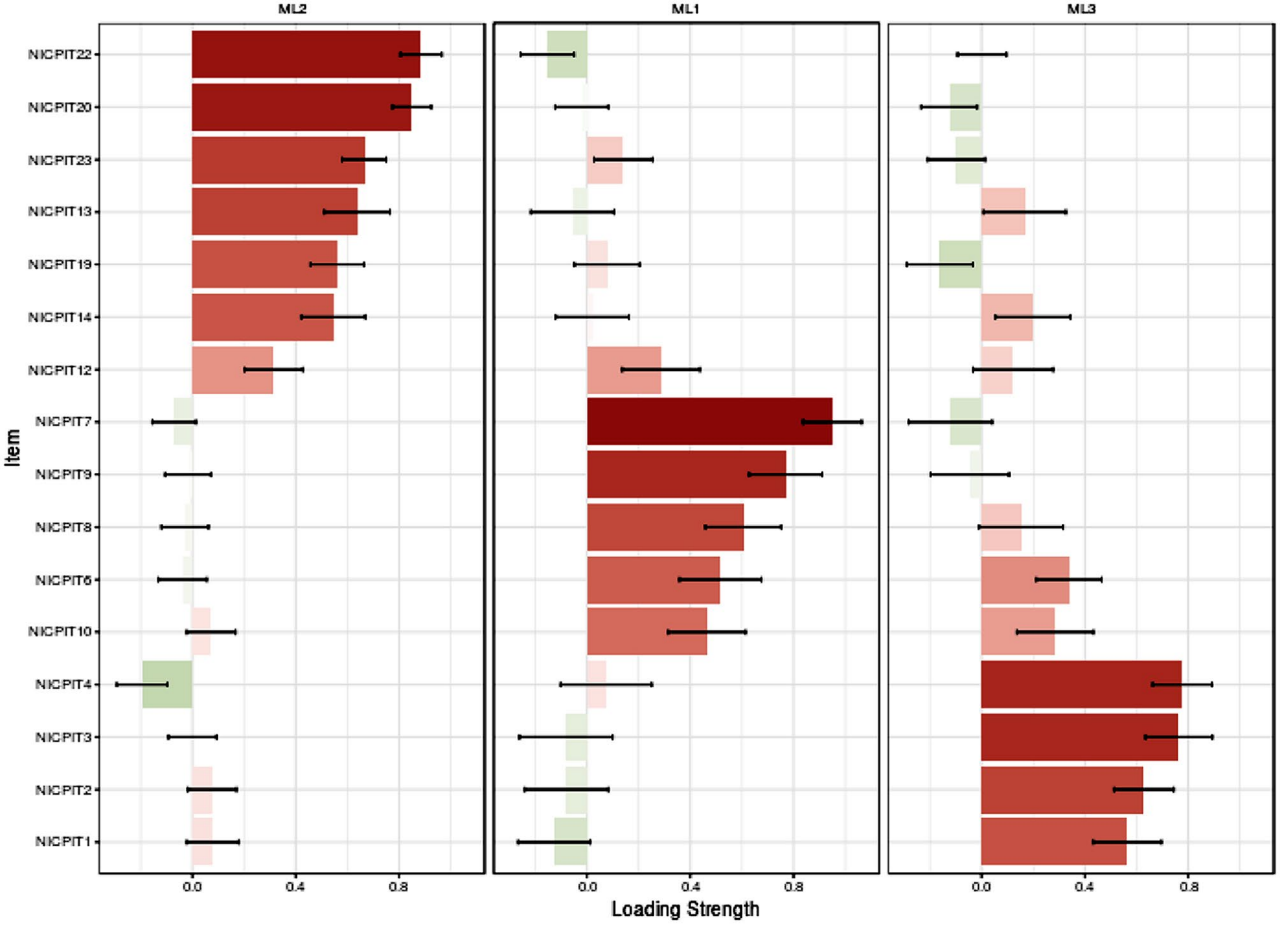
Loading strength of items in factors

In EFA with maximum likelihood method, KMO (0.896) and Bartlett’s value 3595.623 (*p* < 0.001) showed the sample was adequate and suitable. Three factors were discovered using the EGA and parallel analysis. In the EFA, after applying Promax rotation, three factors with a total number of seventeen items were extracted from 21 items. As a result, NICPS with seventeen items were classified into three factors namely “Social support” with seven items, “Spiritual motivation” with six items and “Job satisfaction” with four items. These three factors explained 56.14% of the total variance of intention to care of patients with infectious disease concept in nurses. The first factor explained 24.10% of the total variance, the second factor explained 17.79%, and the third factor explained 14.25% (Table 2; Figs. 2 and 3).

**Table 2:** The result of EFA on NICPS (N = 228)

Factors	Q_n_. Item	Factor loading	h^2^	λ	%Variance
Social support	**21.** The proportionality of the number of nurses per patient and suitable working hours are effective in my motivation to care for infectious patients.	0.972	0.802	4.098	24.10
	**22**. Creating proper job security is effective in my motivation to care for infectious patients.	0.881	0.726		
	**20**. Observance of justice between personnel by the head nurse is effective in my motivation to care for infectious patients.	0.820	0.640		
	**23.** Promotion of the social status and value of the nursing profession in the public opinion with advertisements in the media is effective on my motivation to care for infectious patients.	0.725	0.496		
	**13.** Access to adequate and standard equipment for personal protection increases my motivation to care for infectious patients	0.637	0.457		
	**19.** Financial and spiritual incentives increase my motivation to care of infectious patients.	0.592	0.263		
	**14.** Observing the principles of personal protection and proper hand hygiene by colleagues increases my intention to care of infectious patients.	0.550	0.436		
Spiritual motivation	**7**. I care of the infectious patient because of my sense of philanthropy.	0.911	0.666	3.025	17.79
	**9.** I care of the infectious patient for God’s pleasure.	0.892	0.531		
	**8.** While caring for an infectious patient, I put myself in position of the patient and the patient’s family.	0.725	0.466		
	**10.** I feel valuable in society by caring of infected patients.	0.622	0.515		
	**6.** My work conscience makes me intent to care of infectious patients.	0.512	0.530		
	**12.** It is pleasure for me to recover an infectious patient.	0.442	0.365		
Job satisfaction	**3.** I am satisfied with the experience I get by caring for infectious patient.	0.860	0.520	2.423	14.25
	**4.** The difficulty of working with infectious patients is bearable for me.	0.823	0.513		
	**2.** Satisfaction with my job makes me intent to care of infectious patients.	0.725	0.393		
	**1.** Accepting the nursing profession and its characteristics increases my adaptability to the challenges of caring for the infectious patient.	0.673	0.289		

*Abbreviations*: **h**^**2**^: Communalities; ʎ: Eigenvalue

**Fig. 4:**
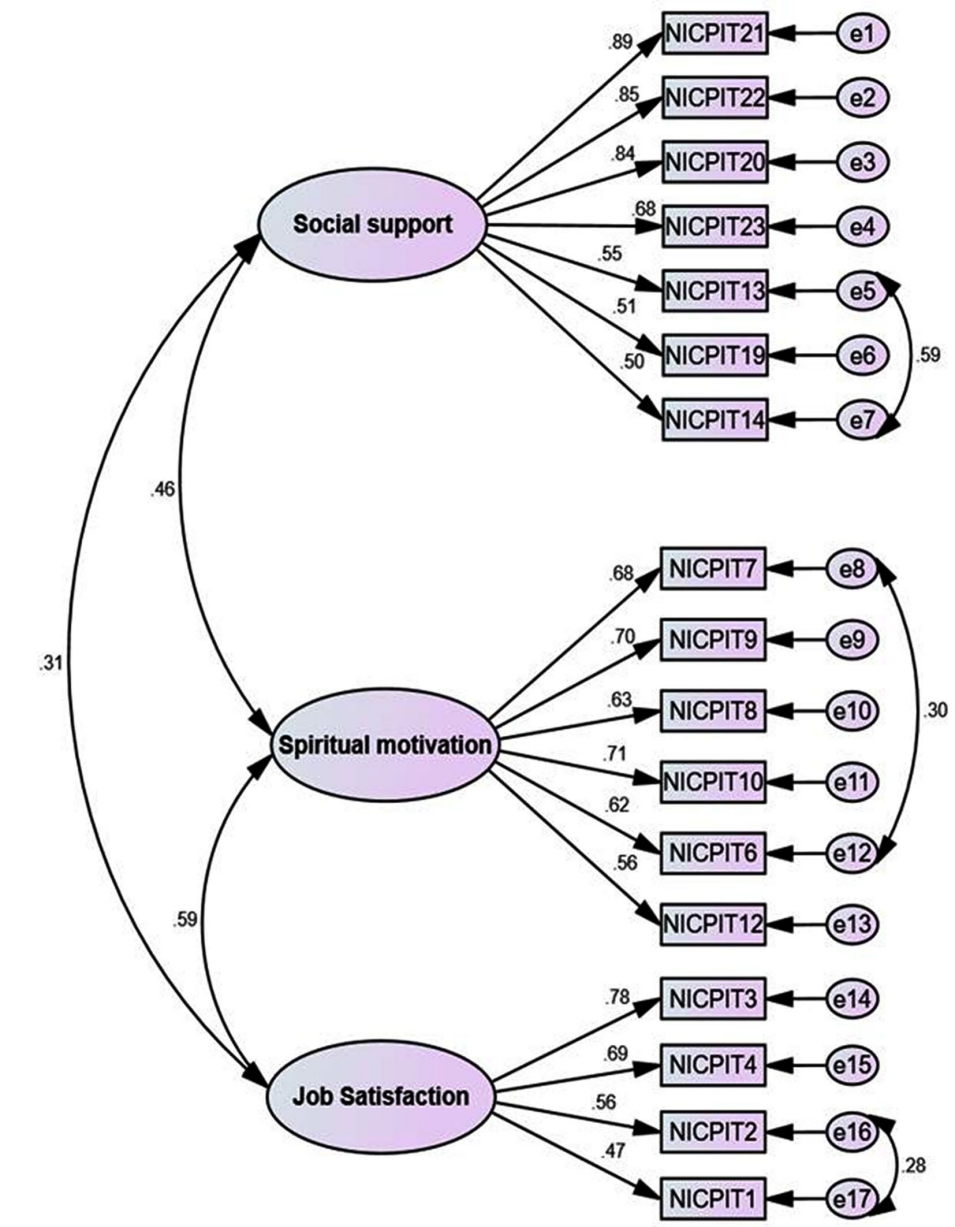
First order CFA of nurses’ intention to care for patients with infectious disease scale (n = 227)

The results of the CFA showed that due to the high regression coefficients (> 0.5), (Fig. 4) in the CFA models as well as the fit indices, the model has a fit and acceptable (TLI = 0.909, CFI = 0.908, IFI = 0.926, PNFI = 0.732, PCFI = 0.768, REMSEA = 0.049, CMIN/DF = 2.477).

Based on the results of CFA, the index values of CR, MSV and AVE ​​showed that the final scale have convergent and discriminant validity in all factors. Furthermore, the results of HTMT confirmed the absence of discriminant validity for the three factors of this scale (Tables 3 and 4).

**Table 3:** The indices of the convergent, discriminant validity, and internal consistency of NICPS of CFA (N = 227)

Factors/index	CR	AVE	MSV	MaxR (H)	α	Ω	AIC
Social support	0.868	0.498	0.215	0.915	0.87	0.87	0.50
Spiritual motivation	0.816	0.426	0.348	0.821	0.84	0.84	0.47
Job satisfaction	0.723	0.404	0.348	0.760	0.74	0.74	0.42

*CR* composite reliability, *AVE* average variance extracted, *MSV* maximum shared squared variance, *Ω* Omega; *α* alpha, *AIC* average inter-item correlation

**Table 4:** The results of HTMT of NICPS

Factors	Support		Spiritual motivation	Job satisfaction
Social support	–			
Spiritual motivation	0.552		–	
Job satisfaction	0.422		0.593	–

According to the results of Cronbach’s alpha (> 0.7), McDonald’s omega (> 0.7), and AIC (≥ 0.4) and MaxR (> 0.7) three factors of this scale have internal consistency (Table 3).

In this study, the ICC value was (0.88, CI 95: 0.87 to 0.89) which showed that the NICPS has strong stability. The absolute reliability based on SEM results was 4.40. This value showed that the score of the scale varies by ± 4.40 in the repeated tests of a person. Based on the results of MDC = 8.71, MIC = 6.35 and LOA: 61.84 to 63.76, this scale has responsiveness and interpretability and there was no ceiling and floor effect.

## Discussion

The results of this study indicated that the nurses’ intention to care for patients with infectious disease concept has seven dimensions including job satisfaction, professional ethics, personal values, standard precautions, preserving health, support, and attitude of patients and their families. Therefore, NICPS is the valid and reliable scale for assessing this concept in nurses.

The current 17-item scale with three factors explained a total of 56.14% of the total variance of the concept of nurses’ intention to care for patients with infectious disease. Since one of the main goals of factor analysis is to maximize variance, the variance of Yoo et al.‘s study was reported as 68.2% [20] and in another study as 53.12% [21].

Based on the results of the internal consistency, the nurses’ intention to care for patients with infectious disease scale is acceptable. The calculation of McDonald’s omega is the advantage of this study because it does not depend on sample size and numbers of items. It is noteworthy that one of the advantages of this scale is having strong stability based on the value of ICC. The value of ICC higher than 0.7 indicates the reliability is favorable [38]. In this study, the value of ICC for the scale was 0.88, which shows the stability of the scale over time.

The first factor of NICPS was labeled “Social support.” Social support is defined as an understanding or experience that a person is liked by others, respected and valued and is part of a social network of mutual assistance and obligations [39]. In this scale, social support was defined as creating job security and adjusting workload and material and spiritual encouragement and providing sufficient equipment for personal protection. The items related to the first factor show that nursing managers supports by rewarding, increase interaction of nurses and facilitate the employment of them. In addition, feeling recognized, appreciated, and respected increases nurses’ satisfaction with their rewards and decreases their intention to leave the job [40]. As a result, social support can increase nurses’ intention to care for patients with infectious diseases.

The second factor was labeled “Spiritual motivation”. Spiritual motivation includes intrinsic motivation and extrinsic motivation, which act as independent motivation in the real sense. Self-motivation is very useful for life and work. Individuals with high spiritual power report more autonomous motivation at work [41]. Inner spirituality, as the most important motivation or driving factor in a person’s life, has a significant impact on the understanding of physical and mental health, social relationships and environmental health. Internal spirituality in the work environment significantly acts as the main motivation of a person [42]. Internal motivation is related to learning, creativity and better performance [43]. In this study, spiritual motivation was defined as internal motivations such as a sense of philanthropy, attention to God’s pleasure, a sense of being valuable in society, and work conscience. The items related to this factor show that nurses’ understanding of spirituality and spiritual care directly affects their performance as well as their relationship with the patient [44]. Therefore, it can affect the intention of nurses to care for patients with infectious diseases.

The final extracted factor was labeled “Job satisfaction”. Job satisfaction is defined as the feeling that a person has towards his job or his job experiences in relation to previous experiences and current expectations [45]. In this study, job satisfaction refers to nurses’ satisfaction with gaining experience, enduring the hardships of work, and adapting to the challenges of caring for infectious patients. The consequences of nurses’ job satisfaction have a significant effect on both nurses and patients [46]. Nursing managers can facilitate the improvement of clinical nursing practice by considering all the characteristics that affect nurses’ job satisfaction.

Another advantage of this study was the evaluation of measurement error, responsiveness, and interpretation of NICPS. So that the results showed, NICPS has the minimum amount of SEM, responsiveness, and interpretability. SEM indicates the accuracy of the measurement for each individual, the lower its value, the higher the reliability [36]. Responsiveness demonstrates the ability of a scale to show changes in a person’s situation over a period. Final, the interpretability shows the ability of the scale to show the meaningfulness of changes. These features are an important and required domain of COSMIN checklist [47]. which has not been reported in the few studies related to the subject of this study.

## Conclusion

The results of present study showed that the NICPS has three factors namely social support, spiritual motivation and job satisfaction. Nursing managers can use these results to provide training and support intervention for nurses in order to increase their intention to care for patients with infectious diseases. Also, the NICPS scale is a reliable and valid scale with 17 items for evaluating the intention to care for patients with infectious disease concept in nurses.

### Limitation

Due to the working conditions of nurses in the infectious wards and the use of masks, gloves and gowns in the department, the interview conditions were difficult. Due to the high workload and lack of time for nurses, data collection was done with difficulty and continuous follow-up. One of the important limitations was concern about the generalization of finding. Because samples were recruited from Iranian populations. Another limitation related to using the online questionnaire for data gathering, therefore it is not possible to verify the participants’ answers due to the lack of physical contact.

### Implication

Understanding the dimensions of the concept of nurses’ intention to care for infectious patients and the use of this scale in different cultures can affect the understanding of nurses’ needs and effective factors in increasing the intention to care and the quality of care for patients with infectious diseases, especially during the outbreak of infectious diseases. Furthermore, the managers of the medical system will not need to be forced to use and provide nurses for these departments. Also, leaving the nursing job will be less. The NICPS with the fewer items and appropriate variance, and being exclusive for nurses’ intention to care for infectious patients is a useful scale for nursing managers and researchers.

### Electronic supplementary material

Below is the link to the electronic supplementary material.

**Supplementary Material 1:** Availability of data and material

## Data Availability

Due to university policies, the datasets generated and utilized for the present study are not publically accessible but are available from the corresponding author upon justifiable request.

## References

[1] DalPezzo NK, editor. Editor nursing care: a concept analysis. Nursing forum. Wiley Online Library; 2009. 10.1111/j.1744-6198.2009.00151.x 19954465

[2] Ghahramanian A, Rassouli M, Zamanzadeh V, Valizadeh L, Asghari E (2020). Good nursing care: Rodgers’ evolutionary concept analysis. Nurs Pract Today.

[3] Karaca A, Durna Z (2019). Patient satisfaction with the quality of nursing care. Nurs open.

[4] Eckhardt M, Hultquist JF, Kaake RM, Hüttenhain R, Krogan NJ. A systems approach to Infectious Disease. Nat Rev Genet. 2020:1–16. 10.1038/s41576-020-0212-5 PMC7839161 32060427

[5] Bloom DE, Cadarette D (2019). Infectious Disease threats in the twenty-first century: strengthening the global response. Front Immunol.

[6] Zandian H, Alipouri-sakha M, Nasiri E, Zahirian Moghadam T. Nursing work intention, stress, and professionalism in response to the COVID-19 outbreak in Iran: a cross-sectional study. Work. 2021(Preprint):1–11. 10.3233/WOR-205099 33867365

[7] Lord H, Loveday C, Moxham L, Fernandez R (2021). Effective communication is key to intensive care nurses’ willingness to provide nursing care amidst the COVID-19 pandemic. Intensive and Critical Care Nursing.

[8] Welt FG, Shah PB, Aronow HD, Bortnick AE, Henry TD, Sherwood MW (2020). Catheterization laboratory considerations during the coronavirus (COVID-19) pandemic: from the ACC’s interventional council and SCAI. J Am Coll Cardiol.

[9] Nicola M, Alsafi Z, Sohrabi C, Kerwan A, Al-Jabir A, Iosifidis C (2020). The socio-economic implications of the coronavirus pandemic (COVID-19): a review. Int J Surg (London England).

[10] Soraci P, Ferrari A, Abbiati FA, Del Fante E, De Pace R, Urso A, Griffiths MD. Validation and psychometric evaluation of the Italian version of the fear of COVID-19 scale. Int J Mental Health Addict. 2020:1–10. 10.1007/s11469-020-00277-1 PMC7198091 32372892

[11] Yan J, Wu C, He C, Lin Y, He S, Du Y (2022). The social support, psychological resilience and quality of life of nurses in Infectious Disease departments in China: A mediated model. J Nurs Adm Manag.

[12] Lee J, Kang SJ (2020). Factors influencing nurses’ intention to care for patients with emerging infectious Diseases: application of the theory of planned behavior. Nurs Health Sci.

[13] Jeong S-a, Kim J (2022). Factors influencing nurses’ intention to care for patients with COVID-19: focusing on positive psychological capital and nursing professionalism. PLoS ONE.

[14] Lee JY, Hong JH, Park EY (2020). Beyond the fear: nurses’ experiences caring for patients with Middle East respiratory syndrome: a phenomenological study. J Clin Nurs.

[15] Catania G, Zanini M, Hayter M, Timmins F, Dasso N, Ottonello G (2021). Lessons from Italian front-line nurses’ experiences during the COVID‐19 pandemic: a qualitative descriptive study. J Nurs Adm Manag.

[16] Lam KK, Hung SYM (2013). Perceptions of emergency nurses during the human swine Influenza outbreak: a qualitative study. Int Emerg Nurs.

[17] Kalateh Sadati A, Zarei L, Shahabi S, Heydari ST, Taheri V, Jiriaei R (2021). Nursing experiences of COVID-19 outbreak in Iran: a qualitative study. Nurs open.

[18] Sharif Nia H, Arslan G, Naghavi N, Sivarajan Froelicher E, Kaveh O, Pahlevan Sharif S, Rahmatpour P (2021). A model of nurses’ intention to care of patients with COVID-19: mediating roles of job satisfaction and organisational commitment. J Clin Nurs.

[19] Bayeh R, Yampolsky MA, Ryder AG. The social lives of infectious Diseases: why culture matters to COVID-19. Front Psychol. 2021:3731. 10.3389/fpsyg.2021.648086 PMC8495420 34630195

[20] Yoo HR, Kwon BE, Jang YS, Youn HK (2005). Validity and reliability of an instrument for predictive nursing intention for SARS patient care. J Korean Acad Nurs.

[21] Rahmatpour P, Sharif Nia H, Sivarajan Froelicher E, Kaveh O, Pahlevan Sharif S, Taghipour B. Psychometric evaluation of Persian Version of nurses’ intention to Care Scale (P-NICS) for patients with covid-19. Int J Gen Med. 2020:515–22. 10.2147/IJGM.S260579 PMC7443411 32884331

[22] Polit DF, Beck CT, Owen SV (2007). Is the CVI an acceptable indicator of content validity? Appraisal and recommendations. Res Nurs Health.

[23] Polit DF, Beck CT. Nursing research: Generating and assessing evidence for nursing practice. Lippincott Williams & Wilkins; 2008.

[24] Lawshe CH (1975). A quantitative approach to content validity. Pers Psychol.

[25] Sharif Nia H, Hosseini L, Ashghali Farahani M, Froelicher ES (2023). Development and validation of care stress management scale in family caregivers for people with Alzheimer: a sequential-exploratory mixed-method study. BMC Geriatr.

[26] Hosseini L, Sharif Nia H, Ashghali Farahani M (2022). Development and psychometric evaluation of family caregivers’ hardiness scale: a sequential-exploratory mixed-method study. Front Psychol.

[27] Widaman KF, Zhang S, Hong S (1999). Sample size in factor analysis. Psychol Methods.

[28] Sharif Nia H, Pahlevan Sharif S, Goudarzian AH, Haghdoost AA, Ebadi A, Soleimani MA (2016). An evaluation of psychometric properties of the Templer’s death anxiety scale-extended among a sample of Iranian chemical warfare veterans. Hayat.

[29] Munro BH. Statistical methods for health care research. lippincott williams & wilkins; 2005.

[30] Rahmatpour P, Peyrovi H, Sharif Nia H (2021). Development and psychometric evaluation of postgraduate nursing student academic satisfaction scale. Nurs Open.

[31] Hosseini L, Froelicher ES, Sharif Nia H, Ashghali Farahani M (2021). Psychometrics of persian version of the 11 items De Jong Gierveld loneliness scale among an Iranian older adults population. BMC Public Health.

[32] Sharif Nia H, She L, Rasiah R, Khoshnavay Fomani F, Kaveh O, Pahlevan Sharif S, Hosseini L (2021). Psychometrics of persian version of the ageism survey among an Iranian older adult population during COVID-19 pandemic. Front Public Health.

[33] Fornell C, Larcker DF (1981). Evaluating structural equation models with unobservable variables and measurement error. J Mark Res.

[34] Henseler J, Ringle CM, Sarstedt M (2015). A new criterion for assessing discriminant validity in variance-based structural equation modeling. J Acad Mark Sci.

[35] Moreno-Jiménez B, Rodríguez-Muñoz A, Hernández EG, Blanco LM (2014). Development and validation of the Occupational Hardiness Questionnaire. Psicothema.

[36] Yuan Y, Kelly P. Absolute validity and test-retest reliability of step counts for Fitbit flex 2. Pram walking: 187: Board# 25 May 29 9: 30 AM-11: 00 AM. Volume 51. Medicine & Science in Sports & Exercise; 2019. p. 36. 6.

[37] Terwee CB, Peipert JD, Chapman R, Lai J-S, Terluin B, Cella D et al. Minimal important change (MIC): a conceptual clarification and systematic review of MIC estimates of PROMIS measures. Uality of Life Research. 2021:1–26. 10.1007/s11136-021-02925-y PMC8481206 34247326

[38] Sharif Nia H, Pahlevan Sharif S, Yaghoobzadehet A, Tahmasbi B, Rassool GH (2018). The Factor Structure of the Spiritual Well-Being Scale in Veterans Experienced Chemical Weapon Exposure.

[39] Taylor SE (2011). Social support: a review. Oxf Handb Health Psychol.

[40] Seitovirta J, Lehtimäki AV, Vehviläinen-Julkunen K, Mitronen L, Kvist T (2018). Registered nurses’ perceptions of rewarding and its significance. J Nurs Adm Manag.

[41] Guillén M, Ferrero I, Hoffman WM (2015). The neglected ethical and spiritual motivations in the workplace. J Bus Ethics.

[42] Nandika SR, Nagalakshmi K (2022). Spirituality as intrinsic motivational factor and health related quality of life among hospitalized male patients practicing Hinduism in India. Industrial Psychiatry Journal.

[43] Wang M, Guo T, Ni Y, Shang S, Tang Z (2019). The effect of spiritual leadership on employee effectiveness: an intrinsic motivation perspective. Front Psychol.

[44] Zakaria Kiaei M, Salehi A, Moosazadeh Nasrabadi A, Whitehead D, Azmal M, Kalhor R, Shah Bahrami E (2015). Spirituality and spiritual care in I ran: nurses’ perceptions and barriers. Int Nurs Rev.

[45] Doran D, Almost J. Nursing sensitive outcomes: the state of the Science. Jones and Bartlett Pub.; 2003.

[46] Liu Y, Aungsuroch Y, Yunibhand J (2016). Job satisfaction in nursing: a concept analysis study. Int Nurs Rev.

[47] Terwee CB, Bot SD, de Boer MR, van der Windt DA, Knol DL, Dekker J (2007). Quality criteria were proposed for measurement properties of health status questionnaires. J Clin Epidemiol.

