# Supplementary material for: Development and psychometric evaluation of nurse’s intention to care for patients with infectious disease scale: an exploratory sequential mixed method study
Source: BMC Nurs. 2024 Jan 24;23:65. doi: 10.1186/s12912-023-01669-z (PMC10807223; doi:10.1186/s12912-023-01669-z)
Supplement: Supplementary file 1 — Supplementary Material 1: Availability of data and material [file 12912_2023_1669_MOESM1_ESM.pdf]

Nurses' intention to care for patients with infectious diseases scale (NICPS).

Qn. Item	Strongly Agree	Agree	Undecided	Disagree	Strongly Disagree
1. The proportionality of the number of nurses per patient and suitable working hours are effective my motivation to care for infectious patients.					
2. Creating proper job security is effective in my motivation to care for infectious patients.					
3. Observance of justice between personnel by the head nurse is effective in my motivation to care for infectious patients.					
4. Promotion of the social status and value of the nursing profession in the public opinion with advertisements in the media is effective on my motivation to care for infectious patients.					
5. Access to adequate and standard equipment for personal protection increases my motivation to care for infectious patients					
6. Financial and spiritual incentives increase my motivation to care of infectious patients.					
7. Observing the principles of personal protection and proper hand hygiene by colleagues increases my intention to care of infectious patients.					
8. I care of the infectious patient because of my sense of philanthropy.					
9. I care of the infectious patient for God's pleasure.					
10. While caring for an infectious patient, I put myself in position of the patient and the patient's family.					
11. I feel valuable in society by caring of infected patients.					
12. My work conscience makes me intent to care of infectious patients.					
13. It is pleasure for me to recover an infectious patient.					
14. I am satisfied with the experience I get by caring for infectious patient.					
15. The difficulty of working with infectious patients is bearable for me.					
16. Satisfaction with my job makes me intent to care of infectious patients.					
17. Accepting the nursing profession and its characteristics increases my adaptability to the challenges of caring for the infectious patient.					
